# Prevalence of Maternal Psychological Distress in pregnant women who receipt prenatal diagnosis of fetal Central Nervous System (CNS) anomalies

**DOI:** 10.1192/j.eurpsy.2022.1579

**Published:** 2022-09-01

**Authors:** C. Falamesca, S. Cappelletti, R. Vicario, P.G. Amante, C. Correale, F. Vigevano, L. Caforio, T. Grimaldi Capitello

**Affiliations:** 1 Bambino Gesù Children Hospital, Clinical Psychology Unit, Neurological Sciences Department, Rome, Italy; 2 Bambino Gesù Children Hospital, Fetal Medicine And Surgery Unit, Department Of Medical And Surgical Neonatology, Rome, Italy; 3 Bambino Gesù Children Hospital, Neurosurgery Unit, Neurological Sciences Department, Rome, Italy; 4 Bambino Gesù Children Hospital, Neurological Sciences Department, Rome, Italy

**Keywords:** Prenatal diagnosis, CNS anomalies, Anxiety, Depression

## Abstract

**Introduction:**

Women receiving a prenatal diagnosis of fetal anomalies are a high-risk population for psychological distress leading to mood disorders. Even so, to date we have no evidence of studies who investigated the levels of maternal anxiety and depression in pregnant women receiving a prenatal diagnosis of fetal CNS anomalies.

**Objectives:**

The aim of this study was to assess the prevalence of anxiety and depression levels in a pilot sample of pregnant women at the Prenatal Diagnostic Unit of the Bambino Gesù Children Hospital.

**Methods:**

We collected data among 43 women who receipt fetal brain anomaly diagnosis (mean age: 35 yrs, SD ± 6.3, range 19-48 yrs; mean week at first access 26w, SD ± 3.9, range 18-33w). Prenatal diagnosis including: ventriculomegaly (37.2%), posterior cranial fossa (23.3%), choroid plexus cysts (11.6%), anomalies of CC (7%) and other (20.9%). Pregnancies with assisted reproductive technology were 14%. We use the Patient Health Questionnaire (PHQ-9) and the Generalized Anxiety Disorders (GAD-7) questionnaires to assess anxious-depressive symptoms.

**Results:**

showed a rate of mild-to-severe anxious depressive symptoms by 60.5% and 48.8% respectively. In detail: 41.9% mild, 14% moderate and 4.7% of severe anxiety. Meanwhile, 41.9% mild and 7% moderate depression. The prevalence of comorbid depressive and anxiety symptoms was 39.5% among the entire sample.

**Conclusions:**

Preliminary data showed a high prevalence of anxious depressive symptoms and comorbidity among pregnant with CNS fetal anomalies. Women receiving a fetal CNS anomaly diagnosis may need additional psychological support or counselling.

**Disclosure:**

No significant relationships.

